# Case report: Clinical and genetic characteristics of heterozygous CaSR variants in three Chinese females with familial hypocalciuric hypercalcemia type 1: a report of three cases

**DOI:** 10.3389/fgene.2025.1570141

**Published:** 2025-05-09

**Authors:** Ruxuan Zhang, Tingting Hu, Shuai Wang, Yiping Cheng, Dandan Luo, Dongmei Zheng, Xinli Zhou

**Affiliations:** ^1^ Department of Endocrinology, Shandong Provincial Hospital, Shandong University, Jinan, China; ^2^ Key Laboratory of Endocrine Glucose & Lipids Metabolism and Brain Aging, Ministry of Education, Jinan, China; ^3^ Department of Endocrinology, Shandong Provincial Hospital Affiliated to Shandong First Medical University, Jinan, Shandong, China

**Keywords:** familial hypocalciuric hypercalcemia, primary hyperparathyroidism, calciumsensing receptor gene, heterozygous variant, parathyroid hormone, cinacalcet

## Abstract

**Background:**

Familial hypocalciuric hypercalcemia (FHH) is an autosomal dominant disorder and represents a rare cause of hypercalcemia. It stems from variants in the calcium-sensing receptor gene (CaSR), G-protein subunit alpha11 gene (GNA11), or adaptor-related protein complex 2 gene (AP2S1), among which variants in the CaSR gene are the most prevalent. However, challenges in the current diagnosis of FHH persist, owing to the overlap in clinical features with primary hyperparathyroidism (PHPT).

**Case presentation:**

The three reported patients demonstrated similar clinical presentations such as hypercalcemia and relative hypocalciuria. In two of them, the parathyroid hormone (PTH) level was elevated, while in one, it was normal. Initially, all of them received conventional hypocalcemic treatment. After comprehensive medical history collection and auxiliary examination were conducted to exclude other causes of hypercalcemia, whole exome sequencing (WES) and sanger sequencing were carried out. The results showed that the three patients carried different variants sites in the CaSR gene, namely, c.887G>A, c.2027 > G, c.1608 + 3A>T and c.332C>T. In addition, c.887G>A was also found in the son and grandson of patient 1. The analysis of the conservation of homologous species and the prediction of protein structure for all variant sites demonstrated that due to the heterozygous variants in CaSR, relatively conserved amino acids were altered, affecting the interaction forces between adjacent amino acids, resulting in changes in the protein structure, which might affect the function of the protein.

**Conclusion:**

In conclusion, we report three cases of FHH1 with different heterozygous variant sites in the CaSR gene. This study has expanded the spectrum of variants. It is of great significance for the genetic screening, diagnosis, counseling, and research of hypercalcemia-related genes and become a key resource for enhancing clinicians’ understanding of FHH1.

## 1 Introduction

FHH is an autosomal dominant condition and constitutes a rare cause of hypercalcemia. In the vast majority of cases, FHH1 is caused by a heterozygous variant of the CaSR gene on the long arm of chromosome 3 (Lee and Shoback, 2018). The rarer forms of FHH, specifically type 2 and 3, are linked to loss-of-function variants in the GNA11 gene and missense variants in the AP2S1 gene, both located on chromosome 19 (Heath et al., 1993; Nava Suarez et al., 2024).

The CaSR is a G-protein-coupled receptor, which is primarily expressed in the parathyroid glands and renal tubules. It plays a crucial role in regulating the secretion of PTH and the excretion of calcium by the kidneys. Inactivation variants of the CaSR gene can result in diminished receptor function, which in turn reduces cellular sensitivity to calcium ions. This alteration may lead to an upregulation of the set point in parathyroid cells and an increase in calcium reabsorption in the kidneys (Christensen et al., 2011; Zahedi et al., 2021). As a result, elevated serum calcium levels may be observed, while urine calcium levels typically remain low to normal. This condition reflects a relative hypocalciuria in the context of concurrent hypercalcemia (Marx, 2015; 2017).

In this study, we describe the clinical features and genetic analysis of three female patients. They were found to have elevated serum calcium levels due to different causes, accompanied by normal or mildly elevated parathyroid hormone levels. After conducting comprehensive medical history collection and auxiliary examination to rule out other causes of hypercalcemia, we performed whole exome sequencing (WES) and sanger sequencing.

To explore the relationship between the variant sites and hypercalcemia, we used bioinformatics software to conduct conservation analysis of homologous species and protein function prediction analysis, further revealing the protein structural changes caused by the missense variants of CaSR. And in combination with previous literature, explore the possible impacts of the above-mentioned variants on the function of the CaSR protein.

## 2 Case presentation

### 2.1 Case1

In June 2021, a 59-year-old female was hospitalized in our department due to elevated serum calcium levels found 8 years ago during a physical examination. Her serum calcium, initially about 2.7 mmol/L (reference range: 2.20–2.70 mmo1/L) with limb weakness, was reduced to normal by self-applied salmon calcitonin nasal spray but recurred 2 years ago during a diabetes-related hospitalization, accompanied by anorexia, insomnia, limb weakness and right femoral pain, fluctuating between 2.80 and 3.12 mmo1/L. The patient had no history of drug use, renal stone disease or parathyroidectomy, and there were no monitored calcium levels in her parents who died of “cerebral infarction” and “esophageal cancer” respectively. Physical examinations were unremarkable. The laboratory investigation revealed hypercalcemia, hypocalciuria, hypernatriuria, hypophosphaturia and normal PTH (Table 1). Other biochemical indexes were normal. Abdominal ultrasound showed a hyperechoic nodule in the right kidney, considering hamartoma. Craniocerebral nuclear magnetic resonance demonstrated a few ischemic foci and maxillary sinusitis. The chest CT showed small nodules and chronic inflammatory changes. Thyroid, parathyroid and cardiac ultrasound showed no obvious abnormalities.

**TABLE 1:** Summary of clinical characteristics.

Varient	Case 1	Case 2	Case 3	Reference range
General information				
Sex	female	female	female	
Age	59	46	59	
Symptoms	anorexia insomnia weakness and femoral pain	nausea and vomiting	-	
medical history	T2DM Cataract, atrial fibrillation, cerebral infarction	congenital eye disease	T2DM	
Biochemical characteristics				
serum calcium (mmo1/L)	2.96	2.85	2.96	2.2–2.7
serum phosphorus (mmo1/L)	1.02	0.63	1.02	0.85–1.51
serum magnesium (mmo1/L)	0.97	1.07	1	0.75–1.02
serum creatinine (μmol/L)	46.4	38.67	42.44	41–81
PTH (pg/mL)	33.68	93.1	136.7	15–65
urine creatinine (mmo1/L)	-	2.99	5.77	-
urine creatinine (mmol/24h)	-	3.59	-	6.2–13.3
urine calcium (mmol/24h)	2.21	1.4	6.24	2.5–7.5
urinary magnesium (mmol/24h)	4.7	0.73	-	3–5
urine sodium (mmol/24h)	312	103.2	145.35	130–240
urine phosphorus (mmol/24h)	6.05	6.23	-	23–48
HbA1c (%)	8.2	-	6.7	4–6
C-peptide (ng/mL)	0.85	-	1.65	1.1–4.4
Triglyceride (mmo1/L)	2.89	1.58	1.36	0.4–1.8
25-hydroxyvitamin D (ng/mL)	7.49	6.56	8.51	lack<10, less than 10–30, equilibrium 30–100, poisoning>100
serum calcium after application of cinacalcet (mmo1/L)	14days:2.62 21day:2.49	7days:2.71 14days:2.51	11day:2.72	2.2–2.7
PTH after application of cinacalcet (pg/mL)	-	7days:75.2	45days:61.84	15–65 (Case3:12–88)

### 2.2 Case2

In September 2024, a 46-year-old female was admitted to our department due to nausea and vomiting for half a year and high serum calcium for more than 2 months. Half a year ago, she had daily nausea and vomiting with gastric-content vomitus, without obvious cause. Examinations conducted at external hospitals showed that serum calcium fluctuated between 2.69 and 2.90 mmo1/L, PTH fluctuated between 71.8 and 83.8 pg/mL (reference range: 15–65 pg/mL), low-density lipoprotein cholesterol 4.26 mmol/L (reference range: 1.00–3.37 mmol/L), and normal other biochemistry. Parathyroid ultrasound showed no abnormalities, thyroid ultrasound showed low-density nodules at the outer and posterior aspect of the lower pole of the left lobe of the thyroid gland, parathyroid single-photon emission computed tomography (SPECT) examination showed no significant abnormalities. Lower limb arteriovenous ultrasound, urinary ultrasound, and bone mineral density measurement showed no significant abnormalities. She was diagnosed with “hyperparathyroidism, hypercalcemia and hyperlipidemia”, salmon calcitonin, diuresis, zoledronic acid, and fluid replacement were given.

After drug withdrawal, the calcium increased again. She came to our hospital for further diagnosis. The patient had congenital exotropia of the left eye, small palpebral fissures, limited movement of the left eye, and adduction. Her father had hypertension and denied any family history of other genetic diseases or tumors. Upon admission, relevant laboratory tests were completed (Table 1), and mild osteoporosis was detected from hand and bilateral humeri radiography. On October, she visited another hospital in Beijing. Bone mineral density testing revealed osteopenia in the lumbar spine, while the bone mineral density of the femoral neck and hip was normal. Parathyroid tomography found a radioactive-increased nodule of unknown nature in the right thyroid lobe, with no hyperfunctioning parathyroid tissue.

### 2.3 Case3

In February 2023, a 59-year-old female was admitted to our department for elevated serum calcium for one and a half years. This patient was initially reported in 2024 by our department (Gao et al., 2024). Laboratory tests indicated hypercalcemia and hypocalciuria (Table 1). The serum calcium levels ranged from 2.72 to 2.96 mmol/L, and the 24-h urinary calcium was 6.24 mmol/24h.

## 3 Genetic analysis

With the patients’ informed consent, blood samples were collected. We extracted genomic DNA from samples. The DNA was then fragmented, ligated with sequencing adapters, amplified, and purified. DNA libraries were constructed through hybridization capture methods. We sequenced these libraries on high-throughput platforms. This approach allowed us to analyze exon regions and adjacent intronic sequences (20 bp flanking regions) of 20,700 human genes across the whole exome, along with the complete mitochondrial genome (16,569 bp). Sequencing data were aligned to the human reference genome hg19 (GRCh37), and we comprehensively evaluated target region coverage depth and base call quality metrics. Our workflow also incorporated the detection of large-fragment copy number variations (CNVs) spanning ≥2 consecutive exons in target genes. CNVs meeting our laboratory’s pre-validated analytical performance thresholds were reported directly. For borderline findings, orthogonal confirmation via qPCR or multiplex ligation-dependent probe amplification (MLPA) was carried out before clinical reporting.

Then we used UniProt and DNAMAN to conduct a conservation analysis of the amino acids at the variant sites across different species.

### 3.1 Case1

**FIGURE 1:**
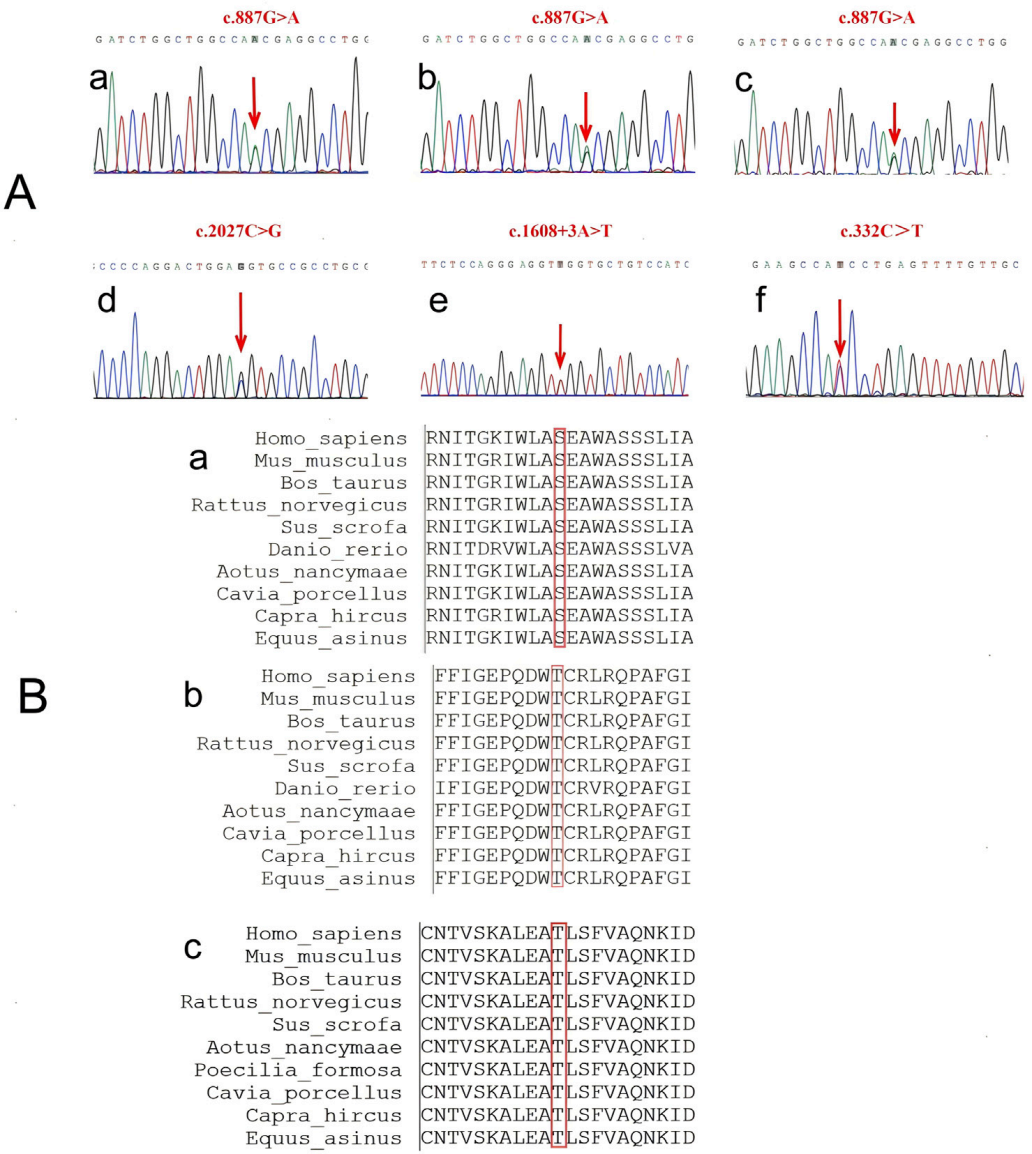
The sequence of the CaSR gene. **(A)** Sanger sequencing results. a-c are the variants in patient one and her son and grandson, all three of them carry the same gene variant, c.887G>A. d-e are two variants in patient 2, c.2027C>G and c.1608 + 3A>T. f is the variant in patient 3, c.332C>T. **(B)** Conservation of amino acids of the CaSR gene among ten species.

The patient one was found to have a heterozygous variant of c.887G>A:p.Ser296Asn (CaSR: NM_000388.4: Exon 4), changing serine to asparagine at amino acid 296 of the encoded protein (Figures 1A, a). Later, we found that her son and grandson who have no obvious clinical symptoms carried the same variant (Figures 1A, b, c).

A cross-species conservation analysis of the variant site showed the amino acid at position 296 was serine in all ten species examined (PhyloP value = 6.131), indicating the amino acid sequence at position 296 of the extracellular domain is relatively conserved (Figures 1B,a).

### 3.2 Case2

The genetic test showed that the subject carried a heterozygous variant of c.2027C>G: p.Thr676Arg (CaSR: NM_000388.4: Exon 7). This variant resulted in a variant from threonine to arginine at amino acid 676 of the encoded protein (Figures 1A, d). Although this variant has been reported four times in ClinVar database, its clinical significance remains conflicting: likely benign (RCV000793144.9) and variants of uncertain clinical significance (VUS) (RCV002290430.3, RCV002477802.1 and RCV004027435.1).

The conservation analysis of the amino acid showed that the amino acid at position 676 is threonine in all ten species (PhyloP value = 3.371). The results show that the amino acid sequence at position 676 of the extracellular domain is relatively conserved (Figures 1B, b).

In addition, this patient carried a heterozygous variant of c.1608 + 3A>T (CaSR: NM_000388.4: Intron 5) (Figures 1A, e), which is located in the non-coding region of the CaSR gene.

### 3.3 Case3

Genetic testing showed that she carried a heterozygous variant of c.332C>T:p.Thr111Ile (CaSR: NM_000388.3: Exon 3), which resulted in a variant from threonine to isoleucine at amino acid 111 of the encoded protein (Figures 1A, f).

We conducted a more comprehensive conservation analysis of the amino acids at the variant sites across various species based on previous studies. It was found that the amino acid at position 111 is threonine in all ten species. A PhyloP value of 5.988 indicates that this site is relatively conserved among different species (Figures 1B, c).

## 4 Variant interpretation

For variant classification, we followed the 2015 American College of Medical Genetics and Genomics (ACMG) guidelines. Variants were classified into three categories: pathogenic, likely pathogenic, or “VUS”.

### 4.1 Case1

The c.2027C>G:p.Thr676Arg variant in the CaSR gene was previously documented in a FHH1 patient (PS4_Supporting; Hannan et al., 2012), though it remains absent from the gnomAD population database (PM2_Supporting). Pathogenicity is further supported by the gene’s elevated missense constraint (gnomAD missense Z-score = 3.123, PP2). Final classification: VUS (PS4_Supporting + PM2_Supporting + PP2).

### 4.2 Case2

The c.2027C>G:p.Thr676Arg variant in the CaSR gene is located within a well-characterized mutational hotspot region (PM1). Supporting this, the HGMD database documents multiple pathogenic missense variants in this region, including c.2039G>A:p.Arg680Cys, c.2043G>T:p.Gln681His, c.2038C>T:p.Arg680Cys and c.2045C > T:p.Pro682Leu. Population frequency data from gnomAD demonstrate this variant’s rarity, with an allele frequency <0.0001 in the general population and 0.000009 in the highest frequency subpopulation (European non-Finnish), with no homozygous cases reported (PM2_Supporting). Computational evidence further supports pathogenicity, as reflected by a REVEL score of 0.842 (Pejaver et al., 2022), suggesting a deleterious effect on CaSR protein function (PP3). Final Classification: VUS (PM1 + PP3 + PM2_Supporting).

SpliceAI analysis predicts that the c.1608 + 3A>T variant alters the donor splice site of intron five in transcript NM_000388.4 (donor loss delta score = 0.35), potentially leading to aberrant splicing (PP3). This prediction gains biological plausibility from the established pathogenicity of c.1608 + 1G>A, a variant at the same splice-donor motif (Di Resta et al., 2014; PS1_Moderate). The variant’s absence from gnomAD (PM2_Supporting) provides additional, albeit limited, evidence for potential clinical significance. Final Classification: VUS (PS1_Moderate + PP3 + PM2_Supporting).

### 4.3 Case3

The c.332C>T:p.Thr111Ile variant has not been previously reported in the gnomAD population database, with its first documentation occurring in our hospital’s 2024 report (PM2_Supporting). Pathogenicity is supported by the gene’s high missense constraint, as evidenced by a missense Z-score of 3.12333, which exceeds the ClinGen threshold of 3.09 (PP2). Additionally, multiple bioinformatics tools (SIFT, PolyPhen-2, and MutationTaster) unanimously predict this variant to be deleterious (PP3). Final Classification: Variant of Uncertain Significance (VUS) (PM2_Supporting + PP2 + PP3).

## 5 Prediction and analysis of protein 3D structure

Although all four variants were classified as “VUS” according to ACMG guidelines, their potential pathogenicity cannot be ruled out, given that all three patients exhibited hypercalcemia unexplained by conventional causes. Notably, the second variant in patient two was located in a non-coding region, we will focus on the remaining other three variants in functional domains for subsequent 3D structural modeling analysis. AlphaFold 2.1 was utilized to conduct in-depth homology modeling of the tertiary structures of both the wild-type and mutant CaSR proteins specific to each of the three patients. Subsequently, Pymol2.2 was employed to render the protein models in a visually interpretable format, enabling a comprehensive analysis of the influence exerted by gene variants on the protein structure.

**FIGURE 2:**
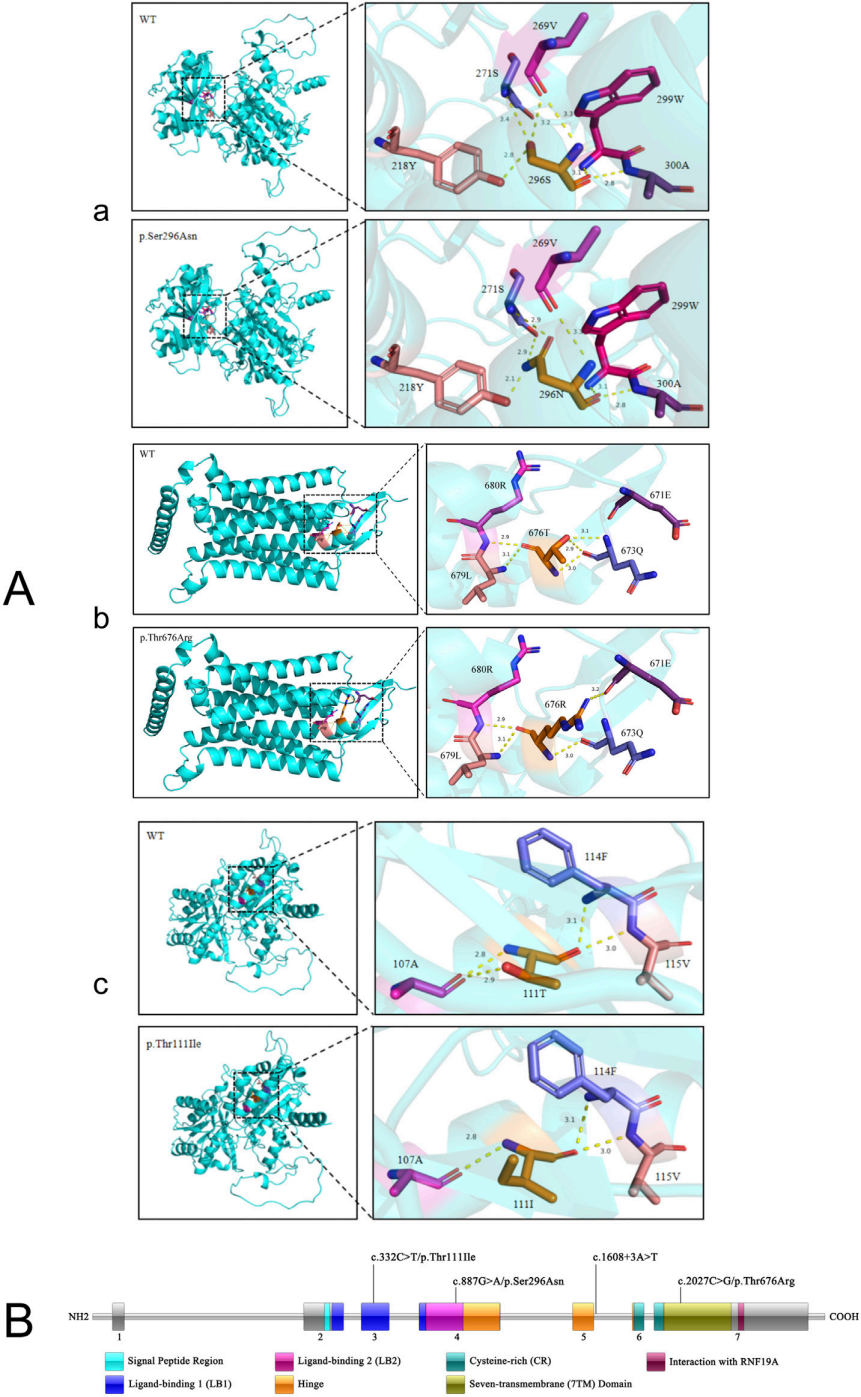
The structure of the CaSR protein. **(A)** Prediction and analysis of protein 3D structure. The modeling was carried out for the mutant-type CaSR protein and wild-type CaSR protein of three patients respectively. **(B)** The specific locations of the four variant sites on the CaSR protein.

In patient 1, the distance of the hydrogen bonds formed between the amino acid at position 296 and the amino acid at position 218 decreases from 2.8 Å to 2.1 Å. Additionally, the hydrogen bonds with the amino acid at position 271 change from one bond at a distance of 3.4 Å to two bonds at a distance of 2.9 Å. Furthermore, the hydrogen bond that previously existed between the amino acid at position 296 and the amino acid at position 269, which had a distance of 3.2 Å, is no longer formed (Figures 2A, a). In patient 2, following the variant, hydrogen bonds with distances of 2.9 Å and 3.1 Å were not established between the amino acids at positions 676 and 673; however, a hydrogen bond at a distance of 3.2 Å was observed from the amino acid at position 671(Figures 2A, b). In patient 3, upon the occurrence of the variant, the structure of the amino acid at position 111 was altered, resulting in the inability to form hydrogen bonds with the amino acid at position 107, which are typically at a distance of 2.9 Å (Figures 2A, c).

These aforementioned changes led to a weakening of the interaction forces between the amino acids, thereby exerting an impact on the three-dimensional structure of the protein in turn.

## 6 Treatment and outcome

At first, all three patients were given conventional calcium-lowering treatments, including hydration, diuresis, zoledronic acid, and salmon calcitonin. Calcimimetic medications, such as cinacalcet hydrochloride, have been proven effective in reducing or even eliminating hypercalcemia in adult and pediatric patients with FHH (Alon and VandeVoorde, 2010; Ritz, 2005). After genetic testing was completed and common causes of hypercalcemia were excluded, cinacalcet tablets (25 mg/d) were administered to all three patients. Subsequently, the dosage was adjusted according to the patients’ blood calcium levels. As a result, the blood calcium levels of all three patients decreased significantly compared with those before the adjustment (Table 1). It's worth noting that cinacalcet commonly causes adverse reactions such as nausea and vomiting. Specifically, patient 2 has experienced relatively severe vomiting. We added gastric-protecting drugs on the basis of using cinacalcet and tried to reduce the dosage of cinacalcet to find the minimum dosage of cinacalcet that can maintain the patient’s normal blood calcium level. In addition, the use of cinacalcet may cause some rare adverse effects, such as low blood pressure, and prolonged QT interval. In the course of use, the patient’s symptoms and related physiological indicators need to be closely monitored (Palmer et al., 2020; Yokoyama et al., 2021; Yousaf and Charytan, 2014).

## 7 Discussion

The overall prevalence of hypercalcemia in the general population is 0.1% (Tonon et al., 2022). Hypercalcemia can be caused by various factors, including hyperparathyroidism, medications, granulomatous disorders, and malignancies. While primary hyperparathyroidism (PHPT) represents the most common etiology of PTH-dependent hypercalcemia, familial hypocalciuric hypercalcemia (FHH) constitutes a distinct genetic disorder (Han et al., 2020; Wang et al., 2020). The classic biochemical abnormalities associated with FHH include moderate hypercalcemia, serum PTH levels that are inappropriately normal or elevated, and relatively low urinary calcium, all while the parathyroid glands appear histologically normal (Mahajan et al., 2020; Mastromatteo et al., 2014). Misdiagnosis as PHPT may lead to unnecessary parathyroidectomy, which fails to correct hypercalcemia in FHH patients.

The majority of patients with FHH1 are asymptomatic and are frequently identified during routine physical examinations or when seeking treatment for unrelated diseases. Those who are symptomatic may experience manifestations such as fatigue, weakness, constipation, polyuria, polydipsia, or headache; a very small subset of patients will also have osteoporosis or pancreatitis simultaneously (Nava Suarez et al., 2024). Owing to the factors elucidated above, the diagnosis and treatment of FHH continue to pose significant challenges. In clinical practice, it is of utmost importance to distinguish FHH from PHPT to avert unnecessary parathyroidectomy that may result from misdiagnosis. This is because, in patients with FHH, calcium levels usually do not decline following surgical intervention.

The consensus guidelines recommend performing a preoperative 24-h urine calcium test to calculate the calcium creatinine clearance ratio (CCCR) as the initial step in the algorithms that differentiate FHH from PHPT and determine who should undergo genetic testing (Eastell et al., 2014). When the CCCR is less than 0.01, the patient is more likely to have FHH, while a value greater than 0.02 usually indicates PHPT (Bletsis et al., 2022). But when CCCR is between 0.01 and 0.02, one-fifth of the individuals with FHH can overlap with PHPT while 4% of cases with PHPT may be misclassified as FHH. In ambiguous cases, further imaging tests and genetic testing assume utmost importance (Christensen et al., 2008; Han et al., 2020). In this manuscript, we report three females with hypercalcemia. Only one patient had complete results of urinary creatinine and 24-h urinary creatinine (Table 1). Therefore, we did not set the CCCR as the main differential criterion in the above text. However, the patients’ parathyroid ultrasound, radionuclide imaging, and genetic testing results could be used to rule out PHPT. Genetic testing was completed for all three patients, and four CaSR gene variant sites at different locations were found (Figure 2B).

All three patients carried distinct variants in the CaSR gene: c.887G>A in patient, c.2027C>G and c.1608 + 3A>T in patient 2, and c.332C>T in patient 3. The second variant (c.1608 + 3A>T) in patient two was located in the intronic non-coding region, and its clinical significance remains unclear. Therefore, subsequent analyses focused on the three missense variants.

The CaSR is a 1078-amino acid G-protein-coupled receptor belonging to family C, including the ECD, transmembrane domains (TMDs) and intracellular domain (ICD). The ECD consists of about 612 amino acids, serves as the binding site of ligands such as Ca^2^+, contains multiple cysteine-rich regions and plays an important role in the correct folding of receptors, dimerization, and ligand binding. The TMDs consist of seven transmembrane helices, which are the key structures for the receptor to transmit extracellular signals to the intracellular. Different transmembrane helices are connected by the intracellular ring and the extracellular ring. The ICD is located in the cell and consists of about 210 amino acids, which can interact with a variety of intracellular signal transduction proteins to initiate downstream signal transduction pathways (Gorvin, 2019; Hannan et al., 2012). CaSR gene variants include missense, nonsense, deletion, insertion and splicing variants, among which missense variants are the most prevalent, accounting for about 85% (Bletsis et al., 2022). The variants in the aforementioned three patients are missense variants. Missense variants affect protein function by altering the three-dimensional structure of proteins, affecting the active site of proteins, interfering with the subcellular localization of proteins, and affecting the stability of proteins. The 3D structure of a protein is maintained by a variety of interactions among amino acid residues, including hydrogen bonds, hydrophobic interactions, van der Waals forces, and ionic bonds. Disruptions to these interactions may occur due to amino acid substitutions caused by missense variants, resulting in alterations to the local structure of the protein, and thus affect the function.

The cross-species conservation analysis demonstrated that these variant sites were completely conserved across 10 homologous species (PhyloP scores >3), suggesting their critical role in maintaining CaSR protein function.

The c.887G>A:p.Ser296Asn variant in patient one was located in the flexible loop region of the extracellular calcium-binding site (CaBS). The serine residue at this position is essential for Ca^2+^ binding due to its phosphorylation potential. The substitution with asparagine introduces a bulkier side chain and eliminates the hydroxyl group, which not only impedes phosphorylation but also increases loop rigidity, hindering Ca^2+^ entry into the binding pocket. This mechanism closely resembles the calcium hyposensitivity phenotype caused by the adjacent p.Glu297Lys mutation (Brachet et al., 2009). Additionally, residue 218 is part of CaBS-1 (Hannan et al., 2012), and the altered hydrogen bonding between residues 296 and 218 may disrupt Venus flytrap domain (VFTD) closure. Rewiring of the hydrogen bond network could also affect protein folding kinetics, leading to misfolding.

The c.2027C>G: p.Thr676Arg variant in patient two occurred in the transmembrane domain (TMD). The introduction of a positively charged arginine with its bulky side chain disrupts hydrophobic packing in the α-helix, potentially interfering with receptor dimerization and downstream G protein-coupled signaling. This is consistent with the loss-of-function mechanism observed with the p.Arg680Gly mutation in the same region (Cole et al., 2009).

The c.332C>T: p.Thr111Ile variant in patient three was located in the N-terminal ligand-binding domain. Substitution of threonine with isoleucine disrupts the hydrogen bond network with valine at position 107. The loss of this hydrogen bond reduces intramolecular interactions, increasing the protein’s free energy and destabilizing its structure, thereby distorting the local β-sheet conformation. Similar N-terminal variants (e.g., p.Arg185Gln) have been shown to impair receptor stability and membrane localization efficiency (Aubert-Mucca et al., 2021). Furthermore, the larger side chain of isoleucine may introduce steric hindrance.

Since most patients with FHH1 are asymptomatic or have mild symptoms, special treatment is generally not required. The CaSR itself is a potential therapeutic target in more symptomatic cases. As mentioned above, cinacalcet can bind to calcium-sensitive receptors on parathyroid cells, increasing the receptor’s sensitivity to extracellular calcium ions. This allows the parathyroid gland to reduce the secretion of PTH when blood calcium levels are relatively low, thereby indirectly affecting serum calcium levels. In clinical application, gastrointestinal side effects of cinacalcet are relatively common. Patient two admitted to our hospital experienced more severe nausea and vomiting during the treatment with cinacalcet. While providing treatment to protect the stomach and relieve vomiting, we reduced the dosage of cinacalcet, and the patient’s symptoms improved significantly. This reminds us that when initially using cinacalcet, we should start with a low dosage, take the medicine after meals, or use it in combination with proton pump inhibitors. In addition to drugs that regulate blood calcium levels, there are other medications available. For patients with bone problems such as osteoporosis, bisphosphonates may be helpful (Koca, 2023). Calcitonin can inhibit the activity of osteoclasts, reduce the release of calcium from bones, and promote the excretion of calcium in urine, thereby lowering blood calcium levels. However, long-term use may lead to resistance to calcitonin, so it is generally used as a short-term adjunct therapy in hypercalcemic crises or when other treatments are ineffective.

In conclusion, this paper reports the clinical and genetic characteristics of three Chinese females with heterozygous variants at different sites of the CaSR gene, accompanied by persistent hypercalcemia, hypocalciuria, and other diverse urinary biochemical abnormalities. Genetic testing plays a vital role in confirming the diagnosis of patients with hypercalcemia with atypical symptoms and facilitating genetic counseling. In addition, this report describes the treatment of three patients and the management of adverse reactions after medication, which also provides some guidance for the clinical treatment of FHH1. However, in our study, only the relatives of patient one underwent relevant genetic testing. Patient two was found to harbor two distinct CaSR variants (c.2027C>G and c.1608 + 3A>T), however, segregation analysis could not be performed due to unavailable parental samples. Additionally, we cannot rule out the possibility that the patients may harbor other undetected genetic mutations beyond current testing capabilities that could contribute to hypercalcemia. Future investigations should include: longitudinal monitoring of serum calcium levels in all available family members; comprehensive genetic screening of relatives to establish potential variant cosegregation patterns; and functional characterization of the identified variants through *in vitro* studies employing CaSR expression vectors coupled with long-read sequencing technologies to precisely determine their molecular consequences. Although the current study’s sample size (n = 3) limits definitive conclusions regarding genotype-phenotype correlations, these preliminary findings warrant validation through multicenter collaborative studies to assess the generalizability of the observed mutation spectrum and associated clinical manifestations.

## Data Availability

The datasets presented in this study can be found in online repositories. The names of the repository/repositories and accession number(s) can be found in the article/supplementary material.
